# Supplementary material for: Long noncoding RNA repertoire and targeting by nuclear exosome, cytoplasmic exonuclease, and RNAi in fission yeast
Source: RNA. 2018 Sep;24(9):1195–213. doi: 10.1261/rna.065524.118 (PMC6097657; doi:10.1261/rna.065524.118)
Supplement: Supplemental Material [file supp_24_9_1195__index.html]

Long noncoding RNA repertoire and targeting by nuclear exosome, cytoplasmic exonuclease, and RNAi in fission yeast — Supplemental Material 

# Long noncoding RNA repertoire and targeting by nuclear exosome, cytoplasmic exonuclease, and RNAi in fission yeast

## Supplemental Material

- Supplemental\_Figures\_S1-S4.pdf
- Supplemental\_Table\_S1.xlsx
- Supplemental\_Table\_S2.xlsx
- Supplemental\_Table\_S3.xlsx
- Supplemental\_Table\_S4.xlsx
- Supplemental\_Table\_S5.xlsx
- Supplemental\_Table\_S6.xlsx
- Supplemental\_Table\_S7.xlsx
- Supplemental\_Table\_S8.xlsx
