# Supplementary material for: Long noncoding RNA repertoire and targeting by nuclear exosome, cytoplasmic exonuclease, and RNAi in fission yeast
Source: RNA. 2018 Sep;24(9):1195–213. doi: 10.1261/rna.065524.118 (PMC6097657; doi:10.1261/rna.065524.118)
Supplement: Supplemental Material [file supp_065524.118_Supplemental_Figures_S1-S4.pdf]

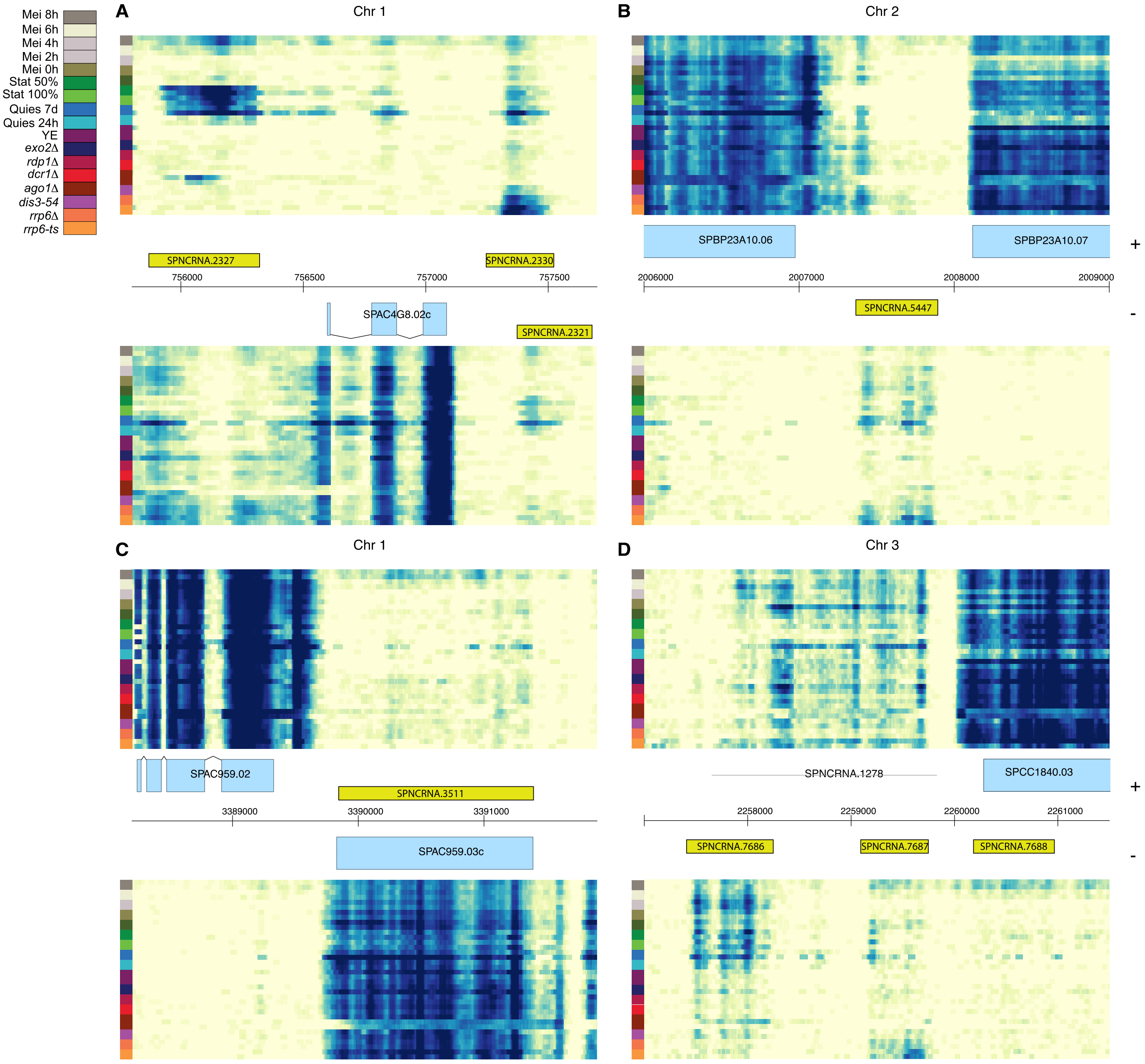

Supplementary Figure S1. Browser view of selected novel lncRNAs (yellow boxes) relative to open reading frames (blue boxes), annotated lncRNA (black line) and novel lncRNAs (yellow boxes), with coordinates indicated in base pairs. Normalised signal intensities (log2 hits per base divided by depth) are shown from blue (high expression) to yellow (low expression) for plus and minus strands as indicated. Selected experimental conditions are presented in rows, colour-coded as indicated at left. Browser view was created using modified R functions from TilingArray package in Bioconductor (Huber et al. 2006, Bioinformatics 22:1963).

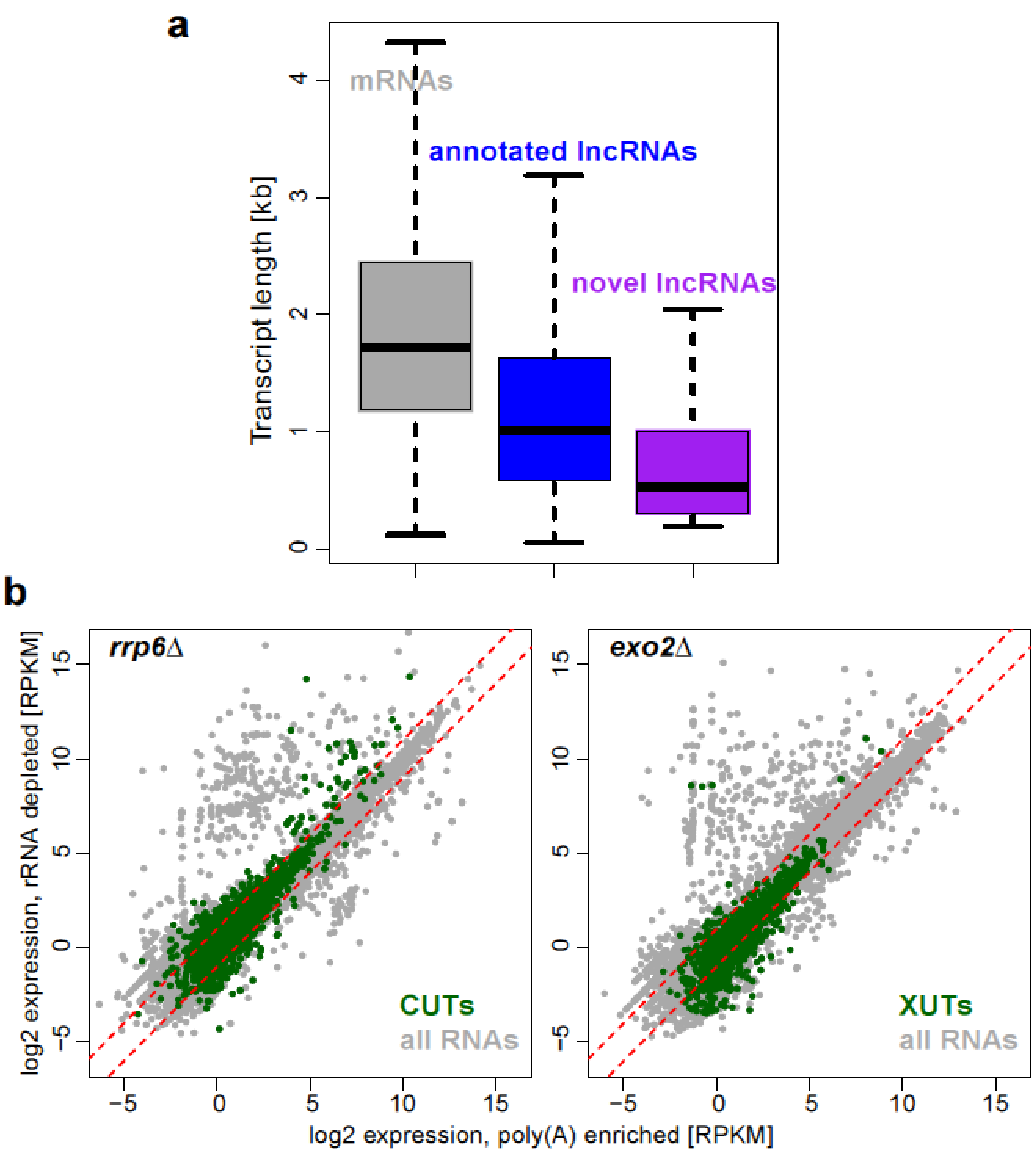

Supplementary Figure S2.
(a) Box plots comparing the transcipt lengths for mRNAs, annotated lncRNAs and novel lncRNAs.
(b) Comparison of RPKM values for samples depleted for rRNAs (Y-axes) vs samples enriched for poly(A) RNAs (X axes). Samples were prepared from *rrp6Δ* (left) or *exo2Δ* (right) mutants, with CUTs and XUTs, respectively, highlighted in green and all other coding and non-coding RNAs shown in grey. Red dotted lines: 2-fold changes. Only 325 and 354 RNAs were induced in rRNA depleted *rrp6Δ* and *exo2Δ* samples, respectively, compared to the corresponding poly(A)-enriched samples. In both cases, 242 of those RNAs are known to lack poly(A) tails, including tRNAs, snRNAs, snoRNAs and mitochondrially-encoded RNAs. Only 49 and 23 novel lncRNAs were induced in rRNA depleted *rrp6Δ* and *exo2Δ* samples, respectively.

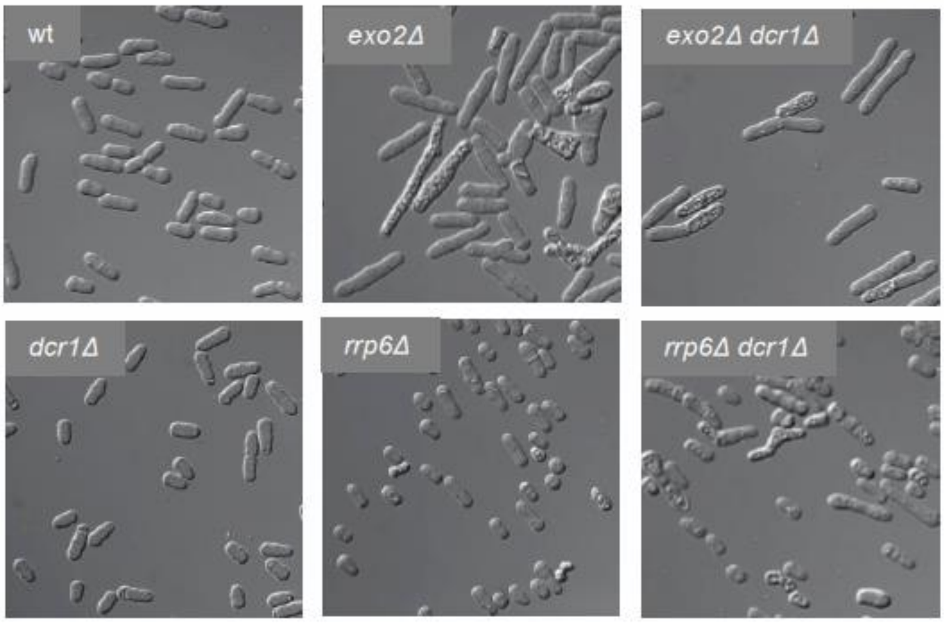

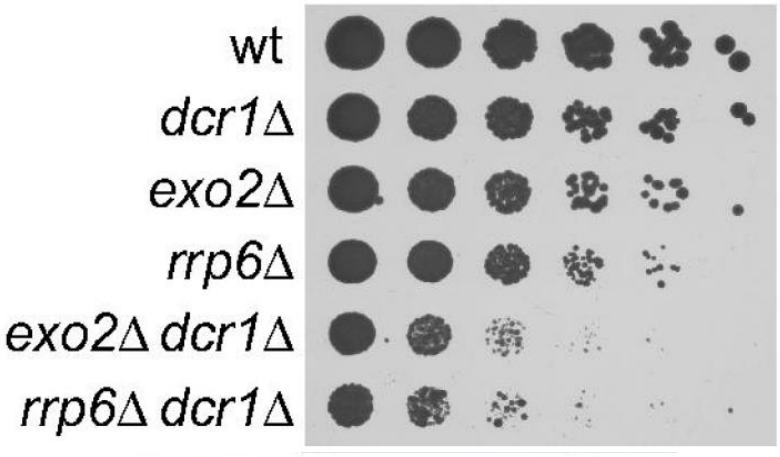

Sample	Maximum growth rate (Arbitrary units)	Average growth rate (Arbitrary units)
WT control	0.29	0.11
dcr1Δ	0.20	0.09
exo2Δ dcr1Δ	0.18	0.08
exo2Δ	0.17	0.07
rrp6Δ	0.11	0.06
rrp6Δ dcr1Δ	0.10	0.05

Supplementary Figure S3. Phenotyping of double mutants.
Top: Live cell micrographs of exponentially growing mutant cells as indicated.
Middle: Six 5-fold serial dilutions of wild-type (wt) and mutant cells as indicated were spotted on YE medium and photographed after 3 days of growth at 32°C.
Bottom: Growth data from liquid cell cultures grown in a BioLector micro-fermentor. Maximum growth rate: biomass change per unit time during exponential phase; average growth rate: average biomass change per unit time during exponential phase. Measurements were taken over a 48-hour period.

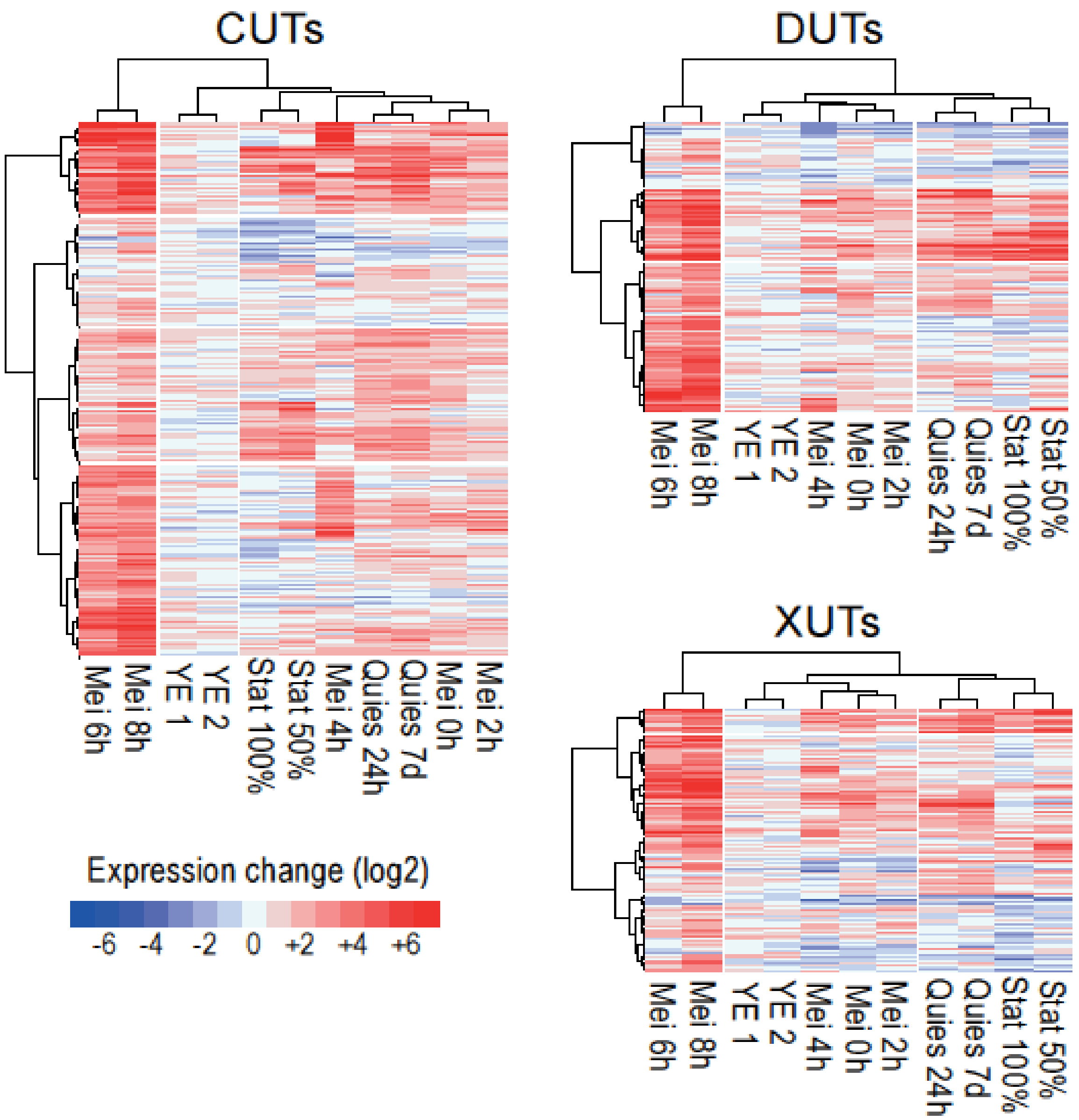

Supplementary Figure S4. Expression changes of CUTs, DUTs and XUTs in different physiological conditions.

Hierarchical clustering of physiological conditions for all CUTs, DUTs and XUTs as indicated. The R pheatmap package was used for clustering applying the Euclidean distance and the ward.D clustering option. Changes in RNA levels in response to the different physiological conditions (indicated at bottom) relative to control cells grown in minimal medium are color-coded as shown in the colour-legend at bottom right (log2 fold-changes). The physiological conditions cluster into three groups: late meiosis (Mei 6-8h), stationary phase (Stat 50-100%), and quiescence/early meiosis (Quies 24h/7d, Mei0-4h).
